# LBSapSal-vaccinated dogs exhibit increased circulating T-lymphocyte subsets (CD4^+^ and CD8^+^) as well as a reduction of parasitism after challenge with *Leishmania infantum* plus salivary gland of *Lutzomyia longipalpis*

**DOI:** 10.1186/1756-3305-7-61

**Published:** 2014-02-07

**Authors:** Rodrigo Dian de Oliveira Aguiar-Soares, Bruno Mendes Roatt, Henrique Gama Ker, Nádia das Dores Moreira, Fernando Augusto Siqueira Mathias, Jamille Mirelle de Oliveira Cardoso, Nelder Figueiredo Gontijo, Oscar Bruna-Romero, Andréa Teixeira-Carvalho, Olindo Assis Martins-Filho, Rodrigo Corrêa-Oliveira, Rodolfo Cordeiro Giunchetti, Alexandre Barbosa Reis

**Affiliations:** 1 Laboratório de Imunopatologia, Núcleo de Pesquisas em Ciências Biológicas/NUPEB, Instituto de Ciências Exatas e Biológicas, Universidade Federal de Ouro Preto, Ouro Preto, Minas Gerais, Brasil; 2 Laboratório de Fisiologia de Insetos Hematófagos, Departamento de Parasitologia, Instituto de Ciências Biológicas, Universidade Federal de Minas Gerais, Belo Horizonte, Minas Gerais, Brasil; 3 Departamento de Microbiologia, Instituto de Ciências Biológicas, Universidade Federal de Minas Gerais, Belo Horizonte, Minas Gerais, Brasil; 4 Laboratório de Biomarcadores de Diagnóstico e Monitoração, Centro de Pesquisas René Rachou, Fundação Oswaldo Cruz-FIOCRUZ, Belo Horizonte, Minas Gerais, Brasil; 5 Laboratório de Imunologia Celular e Molecular, Centro de Pesquisas René Rachou, Fundação Oswaldo Cruz-FIOCRUZ, Belo Horizonte, Minas Gerais, Brasil; 6 Laboratório de Biologia das Interações Celulares, Departamento de Morfologia, Universidade Federal de Minas Gerais, Belo Horizonte, Minas Gerais, Brasil; 7 Departamento de Análises Clínicas, Escola de Farmácia, Universidade Federal de Ouro Preto, Ouro Preto, Minas Gerais, Brasil; 8 Instituto Nacional de Ciência e Tecnologia em Doenças Tropicais/INCT-DT, Salvador, Bahia, Brasil

**Keywords:** LBSapSal vaccine, Canine visceral leishmaniasis, Immunogenicity, Experimental challenge, *Leishmania infantum*, Saliva of *Lutzomyia longipalpis*

## Abstract

**Background:**

The development of a protective vaccine against canine visceral leishmaniasis (CVL) is an alternative approach for interrupting the domestic cycle of *Leishmania infantum*. Given the importance of sand fly salivary proteins as potent immunogens obligatorily co-deposited during transmission of *Leishmania* parasites, their inclusion in an anti-*Leishmania* vaccine has been investigated in the last few decades. In this context, we previously immunized dogs with a vaccine composed of *L. braziliensis* antigens plus saponin as the adjuvant and sand fly salivary gland extract (LBSapSal vaccine). This vaccine elicited an increase in both anti-saliva and anti-*Leishmania* IgG isotypes, higher counts of specific circulating CD8^+^ T cells, and high NO production.

**Methods:**

We investigated the immunogenicity and protective effect of LBSapSal vaccination after intradermal challenge with 1 × 10^7^ late-log-phase *L. infantum* promastigotes in the presence of sand fly saliva of *Lutzomyia longipalpis.* The dogs were followed for up to 885 days after challenge.

**Results:**

The LBSapSal vaccine presents extensive antigenic diversity with persistent humoral and cellular immune responses, indicating resistance against CVL is triggered by high levels of total IgG and its subtypes (IgG1 and IgG2); expansion of circulating CD5^+^, CD4^+^, and CD8^+^ T lymphocytes and is *Leishmania*-specific; and reduction of splenic parasite load.

**Conclusions:**

These results encourage further study of vaccine strategies addressing *Leishmania* antigens in combination with proteins present in the saliva of the vector.

## Background

Dogs are the most important domestic reservoir of *Leishmania infantum*, the protozoan parasite causing visceral leishmaniasis (VL), and represent the major source of contagion for the vector, increasing the risk for human infection [1]. Treatment of infected dogs is not an effective strategy because relapses are frequent, and dogs quickly become infectious again [2]. In this context, a vaccine would be an important tool in the control of canine visceral leishmaniasis (CVL) and would also dramatically decrease the infection pressure of *L. infantum* for humans [3].

Significant efforts are being made by several groups to develop a vaccine against CVL [4-18]. Given their wide spectrum of antigenicity, cost, and safety, the first generation vaccines that composed of crude antigens also represent an excellent tool for immunoprophylaxis [10,11,13-15,19]. In phase I and II clinical trials, Mayrink *et al*. [10], demonstrated enhanced lymphocyte proliferation and significant protection (90%) against experimental infection with *L. infantum* in dogs that had received ultrasound-disrupted, merthiolated promastigotes of *L. braziliensis* with *Bacillus Calmete-Guerin* (BCG). Strong cellular proliferation in response to soluble *Leishmania* antigens has also been reported in dogs vaccinated with autoclaved *L. major* promastigotes plus BCG as the adjuvant [11]. Moreover, in a double-blind randomized efficacy field trial, a single dose of a vaccine composed of alum-precipitated autoclaved *L. major* vaccine against CVL mixed with BCG was shown to be safe and decreased the incidence of the CVL from 12% to 3.7%, which is equivalent to a 69.3% efficacy rate [20].

In the last few decades, the incorporation of salivary proteins of phlebotomines has been widely used in experimental challenge studies, or in seeking potential targets for vaccine development against *Leishmania* infection; such proteins have even been a part of vaccine composition as an adjuvant or co-adjuvant [14,21-29]. Gomes *et al.*[28] showed that hamsters immunized with DNA plasmid coding LJM19 from *Lutzomyia longipalpis* were protected against a challenge with *L. infantum* plus *Lu. longipalpis* salivary gland sonicate [28]. Collin *et al*. [29], working with dogs immunized with saliva recombinant proteins of *Lu. longipalpis* (LJL143 and LJM17) and challenged with uninfected or infected sandflies, observed a cellular immune response at the site of the bite characterized by lymphocytic infiltration and expression of interferon-γ or interleukin-12 [29]. These results suggest that the use of *Lu. longipalpis* saliva proteins could be a good strategy in developing an anti-CVL vaccine in dogs. In this context, previous studies in dogs conducted by our group used a first generation vaccine composed of *L. braziliensis* antigens plus saponin as an adjuvant and sand fly salivary gland extract (SGE) (LBSapSal vaccine). The immunization elicited increases in the anti-saliva and anti-*Leishmania* IgG isotypes, higher counts of circulating and specific T CD8^+^, and high NO production after immunization [14]. The current study included an analysis of the immunogenicity and a parasitological investigation of dogs immunized with LBSapSal vaccine. The dogs were evaluated for up to 885 days after challenge by intradermal inoculation using *L. infantum* promastigotes plus *Lu. longipalpis* SGE.

## Methods

The study protocol was approved by the Ethical Committee for the Use of Experimental Animals at the Universidade Federal de Ouro Preto, Minas Gerais, Brazil.

### Sand flies and salivary gland extracts

Closed colonies of *Lu. longipalpis* were maintained at 25°C and 60%–80% relative humidity according to a published protocol [30]. Sand fly SGE was prepared using the method of Cavalcante *et al.*[31] in which the acini of salivary glands of 4-day-old, mated, but non–blood-fed female sand flies were dissected in 0.8% unbuffered saline, broken by sonication for 10 s, and centrifuged at 10,000 × *g* for 2 min. The supernatant was collected and stored at -70°C prior use.

### Study animals, vaccination, and experimental challenge

In this study, we used the LBSapSal vaccine as previously described by Giunchetti *et al.*[14]. Twenty male and female mongrel dogs were born and reared in the kennels of the Animal Science Center, Universidade Federal de Ouro Preto, Ouro Preto, Minas Gerais, Brazil. The dogs were treated by 7 months of age with an anthelmintic and vaccinated against rabies (Tecpar, Curitiba-PR, Brazil), canine distemper, type 2 adenovirus, coronavirus, parainfluenza, parvovirus, and leptospira (Vanguard® HTLP 5/CV-L; Pfizer Animal Health, New York, NY, USA). The absence of specific anti-*Leishmania* antibodies was confirmed by indirect fluorescence immunoassay and enzyme-linked immunosorbent assay (ELISA) tests. Ouro Preto city is considered a non-endemic area for visceral leishmaniasis in Brazil. Besides negative serology, other additional effective approaches were performed aiming to rule out *Leishmania* infection such as spraying the kennels of UFOP with pyrethroid insecticide and protecting all extension of the kennels with an appropriate and security stainless steel wire mesh to block the access of phlebotomines.

At the beginning of the experiments the dogs were approximately the same age (210 ± 45 days) and had similar weights (15 ± 5 kilograms) and were randomly chosen from a set with approximately the same number of males and females and divided into four experimental groups: (i) the control group C (*n* = 5) that received 1 mL of sterile 0.9% saline; (ii) the Sal group (*n* = 5) that received SGE prepared from five acini of salivary glands of *Lu. longipalpis* in 1 mL of sterile 0.9% saline; (iii) the LBSal group (*n* = 5) that received 600 μg of *L. braziliensis* promastigote protein plus SGE (as above) in 1 mL sterile 0.9% saline; and (iv) the LBSapSal group (*n* = 5) that received 600 μg of *L. braziliensis* promastigote protein plus 1 mg of saponin together with SGE in 1 mL of sterile 0.9% saline. Each group received three subcutaneous injections in the right flank at intervals of 4 weeks. Three and a half months (105 days) after the last vaccine dose, the dogs were challenged via intradermal injection in the right ear, with 1 × 10^7^ late-log-phase *L. infantum* promastigotes (MHOM/BR/1972/BH46) plus SGE obtained from two acini of *Lu. longipalpis* salivary glands [32-34]*.* Dogs were followed for 885 days after challenge. Evaluation of the humoral and cellular immune response was performed before challenge (Tbc; i.e. 85 days before experimental challenge) and 20, 90, 274, 435, 541 and 885 days after challenge (dac). The dogs were euthanized in 885 dac and the spleen was collected to evaluate the parasite load.

### Blood sample collection

Peripheral blood (5 mL) was collected from the jugular vein of each dog and transferred to tubes containing EDTA as anticoagulant. The absolute count of lymphocytes in each sample was obtained using a BC-2800 VET auto hematology analyzer (Mindray, China). Blood samples were stored at room temperature for up to 12 h prior to processing.

### Humoral immune response

The humoral immune response was evaluated by using soluble lysate of *L. infantum* antigen (MHOM/BR/1972/BH46) (SLiA) according to conventional enzyme-linked immunosorbent assay (ELISA) as previously described by Reis *et al*. [35] and Giunchetti *et al*. [13]. Briefly the SLiA was coated onto 96-well microplates (MaxiSorp™, Nalge Nunc Intl., Rochester, NY, USA) at a concentration of 10 μg/well, the serum samples were added at 1:80 dilution, the wells were washed, and peroxidase-conjugated goat anti-dog IgG1 or sheep anti-dog IgG and IgG2 (Bethyl Laboratories Inc., Montgomery, TX, USA) were added at dilutions of 1:1000, 1:8000, and 1:16,000, respectively. The wells were then washed, substrate and chromogen (o-phenylenediamine; Sigma–Aldrich Co., St. Louis, MO, USA) were added, and the absorbance was read at 492 nm on a Multiskan® MCC 340 (Labsystems, Helsinki, Finland) automatic microplate reader.

### In vitro *assays*

For *in vitro* evaluation, peripheral blood mononuclear cells (PBMCs) were isolated from 20 mL of heparinised blood under 10 mL of Ficoll–Hypaque density gradient (Histopaque® 1.077; Sigma Chemical Co.), and centrifuged at 450 × *g* for 40 min at room temperature. The PBMCs were resuspended in Gibco RPMI1640 medium, homogenized, washed twice with RPMI 1640, centrifuged at 450 × *g* for 10 min at room temperature, homogenized, and resuspended in RPMI 1640 at 10^7^ cells/mL. Briefly, for the mitogenic stimulus assays, 25 μL aliquots of PBMCs (2.5 × 10^5^ cells/well) were added to triplicate wells together with 25 μL of phytohemagglutinin (2.5 μg/mL; Sigma-Aldrich Chemie GmbH, Taufkirchen, Germany). Incubations were carried out in a humidified 5% CO_2_ atmosphere at 37°C for 3 days (mitogenic-stimulated cultures) or 5 days (antigenic-stimulated cultures). To investigate the immunophenotypic features, PBMCs were cultured in 48-well flat-bottomed tissue culture plates (Costar, Cambridge, MA, USA) containing 650 μL of supplemented RPMI medium/well. Fifty microliters of PBMCs (5.0 × 10^5^ cells/well) was added to triplicate wells together with 100 μL of vaccine soluble antigen (VSA) (25 μg/mL) or 100 μL of SLcA (25 μg/mL). Incubation was carried out in a humidified 5% CO_2_ atmosphere at 37°C for 5 days, after which the PBMCs were removed for immunophenotyping according to Giunchetti *et al*. [13].

### Immunophenotyping and flow cytometry

Unlabeled canine monoclonal antibodies (mAbs) against CD5 (rat-IgG2a: clone YKIX322.3), CD4 (rat-IgG2a: clone YKIX302.9), and CD8 (rat-IgG1: clone YCATE55.9), all purchased from Serotec (USA), were used in an indirect immunofluorescence procedure in which pooled normal rat serum (diluted 1:6000) was employed as the isotypic control, and fluorescein isothiocyanate (FITC)-labelled IgG sheep anti-rat polyclonal antibody was used as the secondary antibody.

Briefly, microplate assays for immunophenotyping canine whole blood leukocytes in both fresh blood samples and PBMCs obtained after *in vitro* stimulation were carried out according to methods previously described by Giunchetti *et al*. [13]. The results were expressed as the percentage of positive cells within the gated lymphocytes for cell surface markers presenting bimodal distribution (CD5, CD4, and CD8).

### DNA Manipulations, Primers and Real Time PCR of spleen

Total genomic DNA was extracted from approximately 20 milligrams of spleen using Wizard™ Genomic DNA Purification Kit (Promega®, Madison, WI, USA) following manufacturer’s recommendations. To quantify parasite burdens, primers described by Bretagne *et al*. [36] to amplify a 90-bp fragment of a single copy number gene of DNA polymerase of *L. infantum* (GenBank accession number AF009147) were used. PCR was carried out in a final volume of 25 μL containing 200 nM forward and reverse primers, 1×SYBER GREEN reaction master mix (Applied Biosystems, USA), and 5 μL of template DNA. PCR conditions were as follows: an initial denaturation step at 95°C for 10 min followed by 40 cycles of denaturation at 95°C for 15 s and annealing/extension at 60°C for 1 min. Standard curves were prepared for each run using known quantities of pGEM®T plasmids (Promega®, Madison, WI, USA) containing inserts of interest [37]. To verify the integrity of the samples, the same procedure was carried out for the GAPDH gene (115-bp fragment GenBank accession number AB038240). Reactions were processed and analyzed in an ABI Prism 7500-Sequence Detection System (Applied Biosystems, USA). The results were expressed as the number of amastigotes per milligram of spleen.

### Statistical analysis

Statistical analyses were performed using Prism 5.0 software package (Prism Software, Irvine, CA, USA). Normality of the data was detected using a Kolmogorov–Smirnoff test. One-way analysis of variance and Tukey post-tests were used to determine the differences between groups. In all cases, the differences were considered significant when *p* values were < 0.05.

## Results

### LBSapSal elicited lasting production of anti–*L. infantum* immunoglobulin isotypes (IgG, IgG1, and IgG2) after *L. infantum* experimental challenge

**Figure 1:**
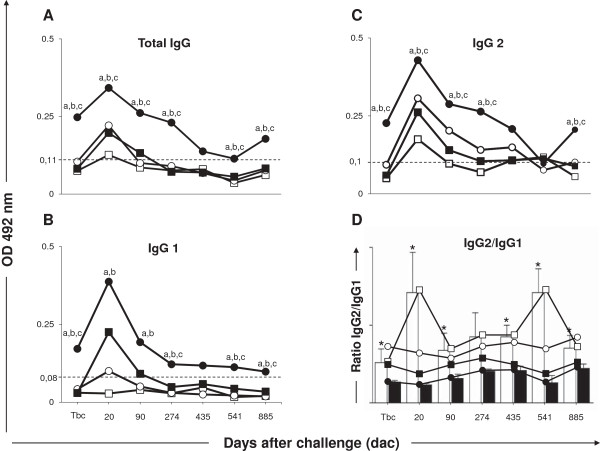
**Anti-*Leishmania *reactivity in serum from dogs submitted to different vaccination protocols before and after intradermal challenge with *L. infantum *plus SGE: C (control;****); Sal (SGE;****); LBSal (killed *L. braziliensis *vaccine plus SGE;****); LBSapSal (killed *L. braziliensis *vaccine plus saponin plus SGE;****). IgG2/IgG1 ratio: C (control;****) and LBSapSal (killed *L. braziliensis *vaccine plus saponin plus SGE;****). (A)** anti–*L. infantum* total IgG; **(B)** anti–*L. infantum* IgG1; **(C)** anti–*L. infantum* IgG2; **(D)** IgG2/IgG1 ratio: C (control; white square) and LBSapSal (killed *L. braziliensis* vaccine plus saponin and SGE; black square), the *x*-axis displays the times at which the assays were conducted (Tbc: time before challenge with *L. infantum*; and 20, 90, 274, 435, 541, and 885 days after challenge with *L. infantum*), and the *y*-axis represents the mean ELISA absorbance values determined at 492 nm in serum samples diluted 1:80 for IgG total and subclasses. The cut-off is represented by the dotted line. Significant differences (*p* < 0.05) between the LBSapSal group and the C, Sal, and LBSal groups are indicated, respectively, by the letters a, b, and c. *Significant difference (*p* < 0.05) between the LBSapSal group and the control C group in IgG2/IgG1 ratio.

Significant (*p* < 0.05) increases in the serum levels of anti-*Leishmania* total IgG at the time before challenge (Tbc; i.e., 85 days before experimental challenge) and 20, 90, 274, 541, and 885 days after challenge (dac) were observed in the LBSapSal group compared with the C, Sal, and LBSal groups (Figure 1A). Also, the LBSapSal group elicited higher levels (*p* < 0.05) of IgG1 at Tbc and 274, 541, and 885 dac compared with the other groups (C, Sal, and LBSal). Moreover, the LBSapSal group showed higher levels (*p* < 0.05) of IgG1 at 20 and 90 dac compared with C and Sal groups (Figure 1B). Higher levels (*p* < 0.05) of anti-*Leishmania* IgG2 were observed in the LBSapSal group at Tbc and 20, 90, 274, and 885 dac compared with other groups (C, Sal, and LBSal) (Figure 1C).

Further analysis demonstrated that the IgG2/IgG1 ratio was lower (*p* < 0.05) in the LBSapSal group compared with C (Tbc and 20, 90, 435, 541, and 885 dac), Sal (Tbc and 20, 90, 435, 541, and 885 dac), and LBSal (Tbc) groups (Figure 1D).

### Increased numbers of circulating T lymphocytes and their subsets were displayed in LBSapSal-vaccinated dogs after *L. infantum* experimental challenge

**Figure 2:**
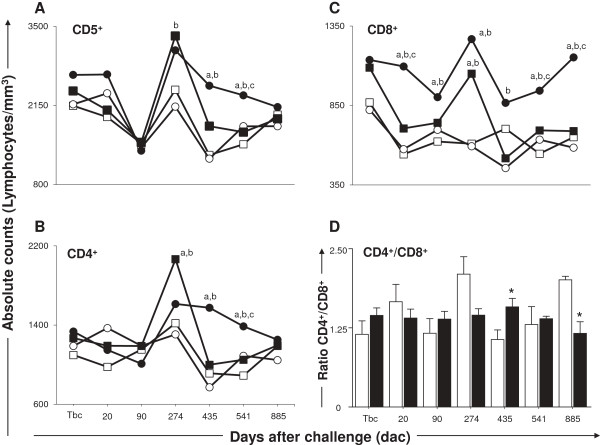
**Cellular profile of circulating lymphocytes in dogs submitted to different vaccination protocols before and after challenge with *L. infantum *plus SGE.** C (control; ); Sal (SGE; ); LBSal (killed *L. braziliensis* vaccine plus SGE; ); LBSapSal (killed *L. braziliensis* vaccine plus saponin plus SGE; ): the *x*-axis displays the times at which the assays were conducted (Tbc: time before challenge with *L. infantum*; and 20, 90, 274, 435, 541, and 885 days after challenge [dac] with *L. infantum*), and the *y*-axis represents the mean values of **(A)** CD5^+^, **(B)** CD4^+^, **(C)** CD8^+^ cells, and **(D)** CD4^+^/CD8^+^ ratio, the white and black bars represented the control and LBSapSal groups, respectively. Significant differences (*p* < 0.05) between the LBSapSal group and the C, Sal, and LBSal groups are indicated, respectively, by the letters a, b, and c. *Significant difference (*p* < 0.05) between the LBSapSal group and the control C group in CD4^+^/CD8^+^ ratio.

In order to evaluate the cellular immunophenotype profile, we enumerated the frequency of T lymphocytes (CD5^+^) and their major subpopulations (CD4^+^ and CD8^+^) (Figure 2). Our results revealed an increase (*p* < 0.05) in the number of circulating CD5^+^ T lymphocytes in dogs vaccinated with LBSapSal group at 435 dac compared with C and Sal groups and at 541 dac when compared with C, Sal, and LBSal groups (Figure 2A).

Analyses of the data showed an increase (*p* < 0.05) of circulating CD4^+^ T lymphocytes in the LBSapSal group compared with the C and Sal groups at 435 dac and the C, Sal, and LBSal groups at 541 dac (Figure 2B). In the same way, higher (*p* < 0.05) CD8^+^ T-cell counts were observed in dogs vaccinated with LBSapSal at 20 dac compared with the C, Sal, and LBSal groups, at 90 and 274 dac compared with the C and Sal groups, at 435 dac compared with the Sal group, and at 541 and 885 dac in comparison with all other groups (Figure 2C). Further analysis demonstrated that the CD4^+^/CD8^+^ ratio was higher (*p* < 0.05) in the LBSapSal group when compared with the C group at 541dac. On the other hand, at 885 dac, the CD4^+^/CD8^+^ ratio was lower (*p* < 0.05) in the LBSapSal group in comparison with the C group (Figure 2D).

### *In vitro* cell proliferation upon antigenic stimuli is persistently increased in LBSapSal-vaccinated dogs after *L. infantum* experimental challenge

**Figure 3:**
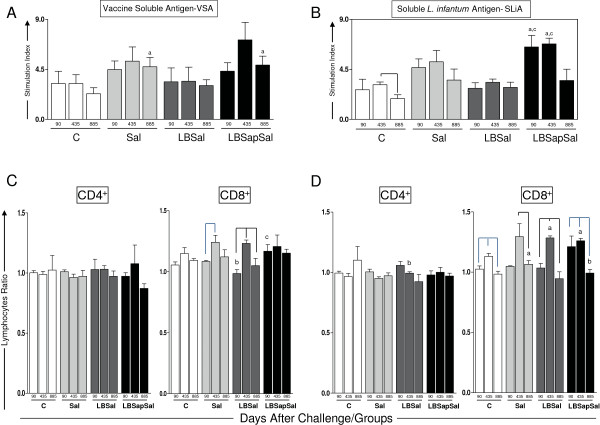
**Cell proliferation response of PBMCs after stimulation with (A) vaccine soluble antigen (VSA) and (B) soluble *L. infantum *antigen (SLiA).** The lower panels show the immunophenotypic profile of *in vitro* PBMCs following stimulation with **(C)** VSA and **(D)** SLiA determined at 90, 435, and 885 dac (days after challenge with *L. infantum* plus SGE) for vaccinated groups: C (control; ); Sal (SGE; ); LBSal (killed *Leishmania braziliensis* vaccine plus SGE; ); LBSapSal (killed *L. braziliensis* vaccine plus saponin plus SGE; ). The results are expressed as the ratio of mean frequencies of CD4^+^ and CD8^+^ cells in the stimulated cultures over non-stimulated cultures (SC/CC). Significant differences (*p* < 0.05) between values measured at 90, 435, and 885 dac under the same group are indicated by connecting lines, and between the LBSapSal and the control C, Sal and LBSal groups are represented by the letters a, b, and c, respectively.

To explore the *in vitro* cell proliferation (PBMCs) we used two different antigenic stimuli: VSA (*L. braziliensis*) in order to evaluate the memory lymphoproliferative immune response against antigens of the vaccine components, and SLiA to investigate possible lymphoproliferative homology with the etiological agent of VL (*L. infantum*) (Figure 3). Comparative analysis of the different treatment groups showed a significantly augmented (*p* < 0.05) stimulation index at 885 dac in the LBSapSal dogs compared with the C (885 dac) groups with VSA stimuli (Figure 3A). In addition, the LBSapSal group exhibited a higher (*p* < 0.05) lymphoproliferative index at 90 and 435 dac compared with the C and LBSal dogs after SLiA stimuli (Figure 3B).

### Higher frequencies of CD4^+^ and CD8^+^ T lymphocytes in antigen-stimulated cultures related to major phenotypic changes in LBSapSal-vaccinated dogs after *L. infantum* experimental challenge

In order to evaluate whether the phenotypic profile of PBMCs in vaccinated/challenged dogs was influenced by VSA or SLiA stimulation, as well as to characterize these cells, we conducted an analysis of the phenotypic features of PBMCs of vaccinated/challenged dogs (Figure 3). In the presence of VSA (Figure 3C), a significant increase (*p* < 0.05) in the stimulated cell/non-stimulated cell ratio of CD8^+^ T cells was observed in the LBSapSal dogs compared with the LBSal group at 90 dac. When we evaluated the SLiA stimulus (Figure 3D), a significant increase (*p* < 0.05) in the ratio of CD8^+^ T cells was observed in the LBSapSal dogs at 90 and 435 dac compared with 885 dac. In addition, the LBSapSal group showed a higher (*p* < 0.05) ratio of CD8^+^ T lymphocytes compared with the C dogs at 435 dac. In contrast, the LBSapSal group displayed a significant decrease (*p* < 0.05) in the CD8^+^ T-cell ratio compared with the Sal dogs at 885 dac.

### Splenic parasite burden is decreased in LBSapSal-vaccinated dogs after *L. infantum* experimental challenge

**Figure 4:**
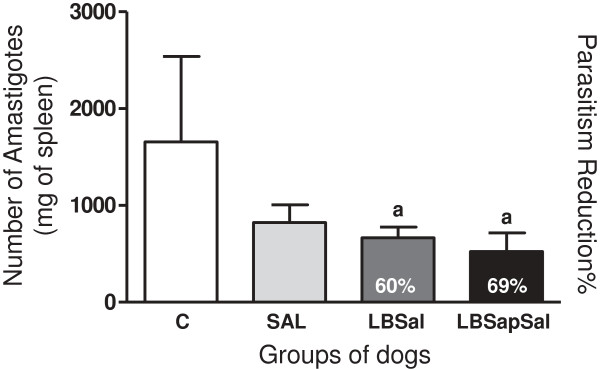
**Quantification of parasite burden in spleen samples at 885 dac (days after challenge with *L. infantum *plus SGE) for vaccinated groups: C (control;****); Sal (SGE;****); LBSal (killed *L. braziliensis *vaccine plus SGE;****); LBSapSal (killed *L. braziliensis *vaccine plus saponin plus SGE; ****).** The *y*-axis displays the quantification of amastigote forms of *Leishmania* per milligram of spleen using real-time PCR with specific primers for a single-copy gene of DNA polymerase of *Leishmania infantum*. The *y*-axis displays inside the respective square of group the parasitism reduction (%) in Sal, LBSal, and LBSapSal in comparison with the C group. Results were plotted representing median values for each group. Significant differences (*p <* 0.05) between the LBSapSal and LBSal groups and the C group are represented by the letter a. C = mean 1657; Sal = mean 823, reduction = 50%; LBSal = mean 667, reduction = 60%; LBSapSal = mean 523, reduction = 69%.

The main parasitological features presented by all animals are summarized in Figure 4. As shown in Figure 4, lower (*p* < 0.05) numbers of amastigotes were observed in the LBSapSal and LBSal groups compared with C dogs. These data are associated with a parasite burden reduction of 60% in LBSal dogs, and 69% in the LBSapSal group compared with C animals. These results indicate the high protective potential of the LBSapSal vaccine even for an extended period after challenge (885 dac).

## Discussion

The addition of sand fly saliva extract in vector-based vaccines can enhance the ability of the host to control or block the parasitic infection [38-41]. In this sense, our study in dogs evaluated the immunogenicity and efficacy of a vaccine composed of *L. braziliensis* crude antigens, saponin as an adjuvant, and sand fly salivary gland extract (LBSapSal vaccine) 885 days after intradermal inoculation using *L. infantum* and SGE of *Lu. longipalpis*.

In a previous study, our group [14] demonstrated that immunization with LBSapSal vaccine can induce high levels of total IgG, IgG1, and IgG2 anti-*Leishmania* antibodies. Interestingly, the present study demonstrated a significant increase of these immunoglobulins 885 days after experimental challenge. It has been proposed that the serum reactivity observed in vaccinated dogs indicates the antigen recognition of *L. infantum*, suggesting the establishment of immunogenic events [13,14,32]. However, it is still not clear which subclass of immunoglobulin would be associated with a CVL resistance pattern. The increases in IgG1 and IgG2 subclasses appear to characterize a mixed profile immune response, as previously described in dogs vaccinated with crude antigens [6,13,14,42,43]. Moreover, there is controversy concerning the association between IgG1 or IgG2 with a profile related to resistance or susceptibility to infection in CVL [44].

Immunophenotyping of canine leukocytes by flow cytometry has been used in an attempt to establish patterns associated with the cellular profile linked to resistance or susceptibility to infection by *L. infantum*[3,45-48], beyond the immunogenic profile in anti-CVL vaccine trials [13,14]. Our results revealed an increase in the number of circulating CD5^+^ T lymphocytes in dogs vaccinated with LBSal and LBSapSal. This increase was associated with high levels of CD4^+^ T- and CD8^+^ T-lymphocyte subpopulations. Interestingly, the LBSapSal vaccine induced a persistent increase in CD8^+^ T lymphocytes throughout the period after challenge. In immunophenotypic CVL studies, increases in CD5^+^ T lymphocytes and subsets of CD4^+^ and CD8^+^ T lymphocytes were reported and related to a profile associated with possible resistance against infection by *L. infantum*[3,49-51]. In a Brazilian endemic area, vaccination of dogs using Leishmune^®^ was associated with increased frequency of CD8^+^ T lymphocytes and an absence of clinical signs 18 months after immunization [8]. The increase in CD8^+^ T circulating lymphocytes in dogs vaccinated against CVL is considered to be an important biomarker of resistance against infection by *Leishmania*[13,14]. Thus, the expansion of CD5^+^ T lymphocytes and the sustained increase of CD4^+^ and CD8^+^ T subsets observed in LBSapSal vaccinated dogs seem to reflect the attempt of the immune system to eradicate the parasite after intradermal experimental challenge. This large antigenic repertoire in the composition of LBSapSal vaccine probably induces responsive memory T lymphocytes, which expand in the peripheral blood inducing a transient increase in CD5 and CD4 lymphocytes, due to exposure to parasite in an attempt of the dogs' immune system to control a possible increase or spread of the *L. infantum* parasite to the organs.

To determine whether the LBSapSal vaccine would be able to activate PBMCs under *in vitro* antigenic stimulation with VSA and SLiA, we measured the stimulation index at 90, 435, and 885 dac in cells derived from immunized dogs. The PBMCs of the Sal and LBSapSal groups were able to recognize and respond to VSA, showing increased stimulation index levels at 885dac compared to the C group. This stimulation observed in PBMC of Sal-vaccinated dogs indicates a nonspecific stimulation and the use of SGE in the experimental challenge could stimulate the immune cells and occasional oscillations occuring in these animals. Moreover, when we stimulated PBMCs with SLiA, an increase in the stimulation index was observed in the LBSapSal group at 90 and 435 dac compared to the C dogs. Interestingly, studies in dogs immunized with a bivalent vaccine composed of crude antigens of *Leishmania* strains (*L. amazonensis* and *L. braziliensis*) associated with BCG as the adjuvant showed greater lymphoproliferative capacity in response to stimulation with antigens of *L. infantum*[15]. Furthermore, the ability to stimulate lymphoproliferative activity using antigens of *Leishmania* has been associated with an immune profile related to resistance to CVL [47,49,50], supporting the hypothesis that the LBSapSal vaccine induces a specific immune response against the causative agent of CVL.

The analysis seeking to identify the phenotypic profile of PBMC after *in vitro* stimulation (VSA or SLiA) showed an increase in the stimulated ratio of CD8^+^ T lymphocytes in the LBSal and LBSapSal groups in the presence of SLiA compared to the C group. In fact, this result reflects the expansion of circulating CD8^+^ T lymphocytes observed in *ex vivo* analysis in the LBSapSal group. In addition, Reis *et al.*[3] described high numbers of CD8^+^ T lymphocytes from the spleens of asymptomatic dogs in the presence of *Leishmania* antigen compared with symptomatic dogs, indicating the importance of these cells to control the *L. infantum* infection in dogs. In this sense, both in humans and dogs, asymptomatic infection has been linked to higher *Leishmania*-specific CD8^+^ T lymphocytes [52,53]. The data from the *in vitro* immunophenotypic profile demonstrates a greater ability of the LBSapSal vaccine to control parasites in dogs [3,47] and reflect improved control of tissue parasitism observed in vaccinated dogs. Thus, the LBSapSal-vaccinated dogs showed a *Leishmania*-specific memory cell consistent with the ability to eliminate parasites after the intradermal experimental challenge.

In the context of the differences between experimental and natural challenges, such as the number of parasites, the life cycle stage (amastigote or promastigote), route of infection, and presence or absence of vector saliva, are important variables that must be taken into account in experimental challenges in the canine model. The experimental challenge using an intravenous route with high numbers of parasites (10^7^–10^8^ promastigotes) seems to be the most efficient challenge model [37]. However, this type of challenge can hide the real experimental vaccine efficacy by suppressing a protective response that could be present in vaccinated dogs [35,54-56]. Thus, an experimental model of infection that most closely resembles *Leishmania* natural transmission is increasingly recommended and desired by many investigators; however, the use of infected sandflies is not easy and remains an obstacle in the dog model. In this work, we used SGE associated with the experimental challenge in an attempt to mimic a sand fly feeding on blood and inoculating an animal with saliva and *Leishmania* promastigotes. However, our results showed that all dogs remained asymptomatic throughout the follow-up (885 dac), indicating that the use of SGE was not sufficient to replicate the bite environment and enhance experimental infection [21-25].

In this study we selected the spleen as the target tissue for detection of the parasite because it is naturally one of the major sites for the parasites [46]. Further, the real-time PCR technique, used by several authors in order to diagnose and screen the evolution of VL in quantifying the parasite burden, offers high sensitivity, accuracy, and reproducibility [36,57,58]. In our work, we applied the real-time PCR technique to quantify the number of amastigotes per milligram of spleen. Interestingly, we found that the LBSal group (60%) and LBSapSal dogs (69%) had a higher proportion of parasite reduction when compared with the control animals. This reduction in parasite load may be associated with the immune response induced by salivary components presenting in both vaccines. Some studies show that *Lu. longipalpis* salivary proteins induce an immune response associated with protection in dogs [14,29]. Furthermore, in a hamster model, salivary protein of a sand fly vector protects against the fatal outcome of visceral leishmaniasis [28]. Thus, the protection obtained in the present study confirms the capacity of this prototype vaccine (LBSapSal) to limit parasite replication even long after challenge (885 days).

## Conclusions

In the present study the LBSapSal vaccine offers great antigenic diversity with persistent humoral and cellular immune responses being elicited, indicating a resistance phenotype against CVL by induction of high levels of total IgG (IgG1 and IgG2); expansion of circulating CD5^+^, CD4^+^, and CD8^+^ T lymphocytes and *Leishmania*-specific; reduction of parasite load in spleen. The results presented in this work encourage further study of vaccine strategies addressing *Leishmania* antigens in combination with proteins present in the saliva of the vector.

## Competing interest

The authors have no conflicts of interest.

## Authors’ contributions

All authors read and approved the final version of the manuscript.
